# Submucous uterine adenosarcoma—minimally invasive treatment

**DOI:** 10.1186/s12957-016-1015-1

**Published:** 2016-10-21

**Authors:** H. Krentel, R. L. De Wilde

**Affiliations:** 1 Clinic of Obstetrics and Gynecology, St. Anna Hospital, Herne, Germany; 2 Clinic of Gynecology, Obstetrics and Gynecological Oncology, University Hospital for Gynecology, Pius-Hospital Oldenburg, Medical Campus University of Oldenburg, Oldenburg, Germany

**Keywords:** Uterine adenosarcoma, Submucous uterine tumour, Gynaecological oncology

## Abstract

**Background:**

Uterine adenosarcomas are rare malignant gynaecological tumours. Due to its submucous localization, they can be easily confound with benign tumours like endometrial polyps or submucous myomas. However, the treatment of uterine adenosarcomas requires an oncologic surgical approach.

**Case presentation:**

In the following case report, we present the minimally invasive treatment of a uterine adenosarcoma by hysteroscopy and laparoscopy in a 37-year-old patient and discuss the special role of hysteroscopy in such cases.

**Conclusions:**

In case of unknown or suspect intrauterine tumours, a diagnostic and operative hysteroscopy with biopsy could be realized prior to laparoscopic hysterectomy especially when the use of a laparoscopic electric morcellation is planned. Thus, a correct oncologic approach can be guaranteed if an adenosarcoma is diagnosed.

**Trial registration:**

ISRCTN

## Background

Uterine adenosarcomas are rare mixed epithelial-mesenchymal tumours and account for approximately 8 % of all uterine sarcomas. Following the WHO classification, they histologically belong to the same group as carcinosarcomas, carcinofibromas and the benign conditions adenofibromas and adenomyomas. Uterine adenosarcomas can be found in all age classes. Gallardo et al. reported a mean patient age of 50 years in 55 cases of adenosarcomas [1]. Most of the adenosarcomas can be found not only in the uterine corpus but also in the cervix, the ovaries, the fallopian tubes, vagina and peritoneum. The uterine tumours clinically appear as submucous polypoid masses with abnormal uterine bleeding and can be easily confound with polyps or myomas in preoperative ultrasound and diagnostic hysteroscopy. Definitive diagnosis of an adenosarcoma is usually achieved only after pathologic examination of the surgical specimen. We present our course of action in a case of a premenopausal patient with a submucous adenosarcoma and discuss the role of diagnostic and operative hysteroscopy in case of uncertain submucous lesions in order to avoid hysterectomy with laparoscopic electric morcellation in adenosarcoma and thus assure an oncologically correct approach in this rare condition.

## Case presentation

**Fig. 1:**
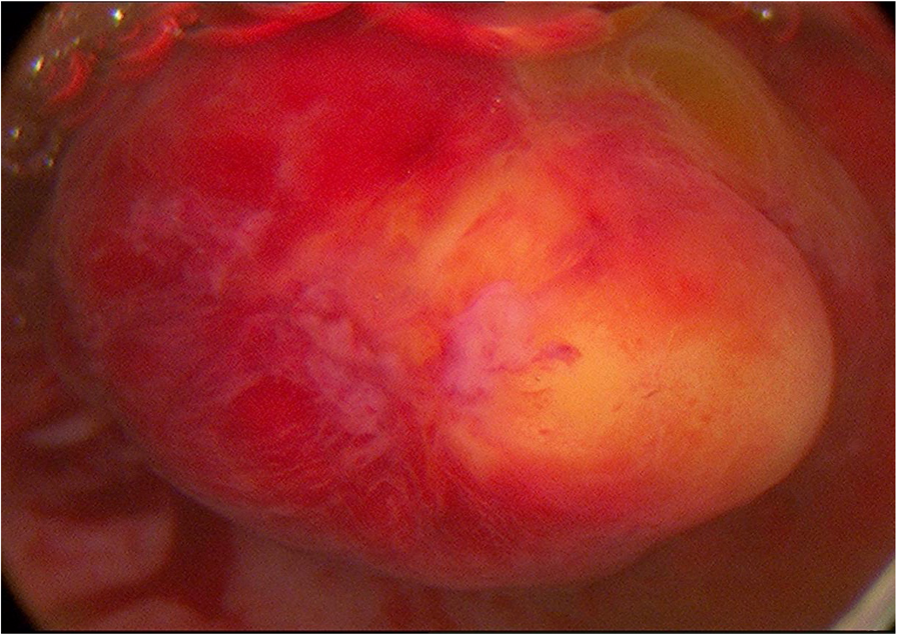
Submucous uterine adenosarcoma in diagnostic hysteroscopy

The 37-year-old patient attended with recurrence of bleeding disorders under oral contraception. The ultrasound examination revealed endometrial hyperplasia. A diagnostic-operative hysteroscopy with uterine abrasion was indicated. The patient gave written informed consent. Hysteroscopy showed a submucous tumour in the left uterine cavity comparable to a submucous polyp or myoma Typ 0 (Fig. 1). We resected the tumour by bipolar hysteroresectoscopy evacuating all tissue fragments from the uterus. The histological examination revealed an epithelial-mesenchymal lesion. Immunohistologically, the epithelial part of the tumour expressed MNF116, while the mesenchymal fraction expressed CD10 and actin. The final diagnosis was an adenosarcoma of the uterine corpus. Although the tumour had been resected completely (R0) by hysteroscopy, we subsequently performed a laparoscopic hysterectomy without power morcellation combined with bilateral salpingectomy, unilateral oophorectomy, a biopsy of the contralateral ovary, peritoneal biopsies and intraperitoneal cytology. No more parts of the adenosarcoma could be found; thus, finally, a low-grade adenosarcoma FIGO stage I was diagnosed. After 4 months, the patient presented with pelvic pain and a cystic ovarian process. Laparoscopy revealed a benign functional ovarian cyst and peritoneal adhesions, but no evidence of recurrence of the adenosarcoma.

### Discussion

Early diagnosis in uterine adenosarcoma is essential because patients’ survival is correlated to the tumour stage. Preoperative physical examination combined with transvaginal ultrasound is not suspicious in most of the cases [2]. Usually, definitive diagnosis of adenosarcoma is achieved only after surgical specimen analysis. Only diagnostic hysteroscopy with intrauterine biopsy or resection allows the histological evaluation of the tumour before definitive hysterectomy and thus a subsequent correct oncologic approach in case of a malignant process. Therefore, especially when hysterectomy with electric morcellation is planned, a hysteroscopy with biopsy could be performed in case of premenopausal submucous lesions in order to differ malignant lesions from benign polyps or myomas. Gonzalez-Bosquet et al. reported one case of hysteroscopic detection of a uterine adenosarcoma in a postmenopausal woman [3] and the diagnosis of an endometrial stromal sarcoma in a 41-year-old woman who presented with bleeding disorders and polypoid masses in ultrasound [4]. Shveiky et al. [5] reported six cases of unexpected malignant uterine mesenchymal tumours. In all cases, the initial hysteroscopic diagnosis erroneously was endometrial polyp or submucous myoma: only pathology revealed the malignancy of the lesions, and thus, all patients underwent complete staging by laparotomy. None of the patients had extrauterine spread of the disease, and at mean follow-up of 21.5 months, all patients were asymptomatic. It plays an important role if the adenosarcoma shows a sarcomatous overgrowth or not. A sarcomatous overgrowth is diagnosed when more than 25 % of the tumour volume is sarcomatous tissue. Gallardo reported that sarcomatous overgrowth was found in 33 % of uterine adenosarcomas. Six out of 29 patients with adenosarcoma developed metastasis. Four of these six patients had adenosarcomas with sarcomatous overgrowth [1]. Schroeder et al. described a systemic therapy in advanced uterine adenosarcoma with sarcomatous overgrowth [6]. Tissue with sarcomatous overgrowth can be detected by immunohistochemical analysis, while the differentiation between adenosarcomas without sarcomatous overgrowth and benign adenofibromas can be difficult. However, a standardized therapeutic approach for adenosarcoma is not yet established. Usually, the treatment is analogue to the therapy of the endometrial stromal sarcoma. In postmenopausal women, the hysterectomy with bilateral adnexectomy and peritoneal staging is the treatment of first choice. In comparison, in premenopausal women, it is possible to save the ovaries. The lymphadenectomy is not established, due to the low rate of lymphatic metastasis. In addition, the lymphatic node status would have no consequence for the adjuvant treatment, as a specific chemotherapy does not exist. Clement et al. reported that radiotherapy has no benefit for the patient in case of adenosarcomas [7]. However, Kaku et al. described that in 31 % of all cases, an extrauterine manifestation of the tumour can be found in the vagina, the lymphatic nodes, the peritoneal cytology or the ovary [8]. In this case of low-grade adenosarcoma without adenosarcomatous overgrowth, we preferred the minimally invasive approach for hysterectomy and staging, as laparotomy has many well-known disadvantages but no benefit in order to carry out a simple hysterectomy with adnexectomy and peritoneal staging. Further studies must show if there is a difference in the prognosis of the disease comparing laparoscopy to laparotomy when staging is realized. It also remains unclear if bilateral adnexectomy should be part of the surgical staging in uterine adenosarcoma. However, in low-grade and high-grade endometrial stromal sarcoma, the bilateral adnexectomy is recommended [9], but ovary-sparing procedures could be considered for young women [10]. In this case of a premenopausal woman, we decided to preserve one ovary and thus the hormonal function. A representative laparoscopic ovarian biopsy did not show any remaining microscopic disease. A second-look laparoscopy after 4 month for ovarian tumour revealed a benign functional cyst.

The main risk factors for recurrence and metastasis in uterine sarcomas are the myometrial infiltration, the tumour size, the extrauterine appearance, the histological tumour type and the presence of sarcomatous overgrowth [8]. The recurrence rate in adenosarcoma without sarcomatous overgrowth is estimated to be 15–25 % respectively and 45–70 % in cases with sarcomatous overgrowth. On a multivariate analysis, Carroll et al. [11] showed that sarcomatous overgrowth and lymphovascular space invasion were predictors of worse progression-free survival and overall survival. Another important prognostic factor is the complete resection (R0) of the sarcoma without intra-abdominal morcellation of the sarcoma. In modern gynaecological minimally invasive surgery, the electric morcellation of uterine tissue in laparoscopic myomectomy or total and subtotal hysterectomy plays an important technical role. The risk of accidental morcellation of occult sarcoma is currently under discussion [12–14]. The incidence of this rare complication has to be shown by further studies but is estimated to be under 1 %. The steering committee on fibroid morcellation of the European Society of Gyneacological Endoscopy (ESGE) concluded that the prevalence of uterine sarcoma in presumed fibroids is 0.14 % with a range from 0.49 to 0.014 % [15]. However, the patients should be informed about this risk when the use of morcellation is planned and alternative surgical approaches should be discussed. While the intraperitoneal morcellation of submucous uterine sarcomas influences the prognosis of the disease, the role of intrauterine biopsy or resection of adenosarcoma in hysteroresectoscopy on the prognosis of the disease so far remains unclear. In comparison, in endometrial cancer, the diagnostic hysteroscopy with biopsy or curettage is a standard procedure and the eventual diversion of cancer cells with the distension medium through the fallopian tubes to the peritoneal cavity is not seen as a risk factor [16]. As adenosarcomas are rare tumours, it will be difficult to achieve reliable prospective data in studies. A second-look laparoscopy after 6 to 12 months could be an option in order to offer the maximal surgical safety to the patient. It is very important to avoid perforation of the uterine wall in the diagnostic or operative hysteroscopy as a peritoneal metastasis could be the result in case of a malignant lesion.

## Conclusions

Submucous adenosarcomas in premenopausal women are rare malignant tumours and can be easily confound with benign endometrial polyps or submucous myomas. A presurgical differentiation by ultrasound is not possible. In order to avoid an oncologically incorrect approach, a diagnostic hysteroscopy with possible consecutive biopsy could be performed in case of submucous uterine tumours when hysterectomy with electric morcellation is planned. Further studies could show if the intrauterine morcellation by hysteroresectoscopy of submucous adenosarcomas has any influence on the prognosis of the disease due to the possible diversion of sarcomatous cells to the abdominal cavity.

## References

[1] Gallardo A, Prat J (2009). Mullerian adenosarcoma: a clinicopathologic and immunohistochemical study of 55 cases challenging the existence of adenofibroma. Am J Surg Pathol.

[2] Leung F, Terzibachian JJ, Aouar Z, Govyadovskiy A, Lassabe C (2008). Uterine sarcomas: clinical and histopathological aspects. Report on 15 cases. Gynecol Obstet Fertil.

[3] Gonzalez Bosquet E, Sunol M, Callejo J, Lailla JM (2005). Uterine adenosarcoma diagnosed following hysteroscopic resection of an intrauterine tumour. Eur J Gynaecol Oncol.

[4] Gonzalez Bosquet E, Sunol M, Cortes L, Murcia N, Callejo J, Lailla JM (2010). Hysteroscopic diagnosis of a high-grade endometrial sarcoma in a 41-year-old woman. Eur J Gynaecol Oncol.

[5] Shveiky D, Revel A, Rojansky N, Benshushan A, Shushan A (2005). Diagnosis of malignant mesenchymal uterine tumors by hysteroscopic excisional biopsy. J Minim Invasive Gynecol.

[6] Schroeder B, Pollack SM, Jones RL (2014). Systemic therapy in advanced uterine adenosarcoma with saromatous overgrowth. Gynecol Oncol.

[7] Clement PB, Scully RE (1990). Mullerian adenosarcoma of the uterus: a clinicopathologic analysis of 100 cases with a review of the literature. Hum Pathol.

[8] Kaku T, Silverberg SG, Major FJ, Miller A, Fetter B, Brady MF (1992). Adenosarcoma of the uterus: a gynecologic oncology group clinicopathologic study of 31 cases. Int J Gynecol Pathol.

[9] Einstein MH, Barakat RR, Chi DS, Sonoda Y, Alektiar KM, Hensley ML, Abu-Rustum NR (2008). Management of uterine malignancy found incidentally after supracervical hysterectomy and uterine morcellation for presumed benign disease. Int J Gynecol Cancer.

[10] Bai H, Yang J, Cao D, Huang H, Xiang Y, Wu M, Cui Q, Chen J, Lang J, Shen K (2014). Ovary and uterus-sparing procedures for low-grade endometrial stromal sarcoma: a retrospective study of 153 cases. Gynecol Oncol.

[11] Carroll A, Ramirez PT, Westin SN, Soliman PT, Munsell MF, Nick AM, Schmeler KM, Klopp AH, Fleming ND (2014). Uterine adenosarcoma: an analysis on management, outcomes and risk factors for recurrence. Gynecol Oncol.

[12] McCarthy M (2014). US agency warns against morcellation in hysterectomies and myomectomies. BMJ.

[13] Tanos V, Brölmann H, De Wilde RL, O’Donovan P, Campo R. Myoma morcellation and sarcoma panic. Gyn Surg. 2014; doi: 10.1007/s10397-014-0876-y.

[14] De Wilde RL, Bojahr B (2014). Intraabdominal sarcoma spread risk due to morcellation in minimal-invasive myoma surgery. Int J Reprod Fertil Sex Health.

[15] Brölmann H, Tanos V, Grimbizis G, Ind T, Philips K, Van den Bosch T, Sawalhe S, Van den Haak L, Jansen FW, Pijnenborg J, Taran FA, Brucker S, Wattiez A, Campo R, O’Donovan P, De Wilde RL (2015). Options on fibroid morcellation: a literature review. Gynecol Surg.

[16] Brandner P, Neis KJ (2000). Diagnosis of endometrial cancer and its precursors. Contrib Gynecol Obstet.

